# Enhancing Conjunctival Vasculature Imaging: A Multi-Objective Cuckoo Search Approach for Contrast Enhancement Optimization

**DOI:** 10.1167/tvst.14.9.36

**Published:** 2025-09-26

**Authors:** Clara Llorens-Quintana, Umut Kuran, Emre Can Kuran, David Madrid-Costa

**Affiliations:** 1 Department of Optometry and Vision, Faculty of Optics and Optometry, Complutense University of Madrid, Madrid, Spain; 2 Department of Computer Engineering, Faculty of Engineering, Harran University, Şanlıurfa, Turkey; 3 Department of Software Engineering, Faculty of Engineering and Natural Sciences, Bandirma Onyedi Eylul University, Bandirma, Turkey

**Keywords:** conjunctival vasculature, image enhancement, image analysis, conjunctival imaging, contrast optimization

## Abstract

**Purpose:**

To develop and evaluate an automated method for enhancing the quality of vascular conjunctival images through optimized contrast and reduced noise.

**Methods:**

Conjunctival images were acquired using a functional slit lamp biomicroscope. The visibility of the vascular structures was enhanced using contrast limited adaptive histogram equalization (CLAHE). The multi-objective cuckoo search (MOCS) optimization algorithm was implemented to tune CLAHE hyperparameters with two objective functions that maximize image contrast and minimize noise amplification. All the images were enhanced using CLAHE with optimized parameters (MOCS-CLAHE) and with predetermined parameters (CLAHE). The performance of both approaches was evaluated through qualitative assessment and quantitative image quality metrics, including peak signal-to-noise ratio (PSNR), structural similarity index (SSIM), and natural image quality evaluator (NIQE).

**Results:**

Both approaches significantly increased the contrast of the original conjunctival images. Despite CLAHE generating images with higher vessel contrast than MOCS-CLAHE, it increases image noise significantly. The overall vessel visibility and quality of enhanced images was significantly better with MOCS-CLAHE, consistently giving higher PSNR and SSIM, and lower NIQE compared to CLAHE.

**Conclusions:**

MOCS optimization is an efficient method to estimate CLAHE parameters when preprocessing conjunctival images acquired with a slit lamp. It provides high quality images of the conjunctival vasculature emphasizing vessels structure by increasing contrast and keeping noise amplification to a minimum.

**Translational Relevance:**

This automated enhancement technique may improve subsequent image segmentation and classification tasks in conjunctival imaging by optimizing contrast and noise reduction for each individual image, thus contributing to more reliable and efficient diagnostic procedures.

## Introduction

The ocular bulbar conjunctiva has a dense vascular network whose conjunctival vessels are branches from the ophthalmic artery. This artery is supplied by the internal carotid artery, and thus conjunctival vessels respond to pathological alterations in ocular, systemic, and central nervous system diseases.^1^^,^^2^ Notably, the conjunctiva is the only part of the body where superficial constituent blood vessels and microcirculation are clearly observed and directly accessible with noninvasive techniques. These unique characteristics make the conjunctival vascular network a perfect emergent target for the diagnosis of diseases with associated vascular alterations. In patients with diabetic retinopathy there are conjunctival alterations that parallel the vascular alterations at a retinal level, but compared to retinal microvessels, conjunctival vessels are easily visible and accessible.^3^ Therefore the study of conjunctival microvessels could provide important information on the onset, progression, and prognosis of diabetic retinopathy in a quick and accessible way.^4^

Different imaging techniques have been used to image the vascular network of the conjunctiva.^5^ Regardless of the technique, conjunctival images often suffer from low contrast, uneven illumination, poor exposure, motion artifacts, or defocus, causing local image degradation and making further computer vision tasks such as automatic vessels segmentation or image classification an arduous task. Additionally, small movements of the subject being imaged translate into large light changes at the image plane, and some instruments are difficult to operate, being tough for the operator to fine-adjust the illumination so fast. It is very frequent to find conjunctival images with poor exposure, which considerably deteriorates the contrast of the image.^6^ Therefore robust enhancement methods are required to increase the visibility of the structure of the vascular network and maximize the information content of the images.

Histogram equalization (HE) approaches are commonly used to enhance the contrast of medical images.^7^^–^^10^ In HE the gray levels of an image are remapped and matched to flat probability density function using a transformation function.^7^^,^^11^ Derived from the most basic approach of global HE, there are other variants that perform local contrast enhancement adapting the transformation function to the local features of the image, like the contrast limited adaptive histogram equalization (CLAHE), which has shown to perform better than the global HE.^12^^–^^14^ In CLAHE, the image is divided into sub-regions in which HE is individually applied. To prevent over enhancement, in each of these sub-regions the histogram of the original image is clipped to a predefined limit and the values above this limit are equally redistributed along all the gray level values before the transformation function is applied. The main advantages of CLAHE over HE is that it prevents noise amplification and over enhancement. The main limitation of CLAHE is that its performance is highly dependent on the choice of the number of sub-regions and the clip limit value. The inaccurate selection of these parameters leads to poor enhancement outcomes and a significant decrease in image quality. Usually the number of sub-regions and the clip limit are predetermined empirically and specifically for a given type of image, making it challenging to automate the process when the input images have different content since it is unlikely that fixed parameters work well for all images.^11^ Joseph et al.^15^ showed the repercussion on image quality of variations of clip limit, tile size, and shape of the matched histogram using magnetic resonance (MR) images and evidenced the degradation in image quality when these parameters are not properly chosen.

The use of an automated algorithm to optimize the parameter selection for CLAHE would allow to improve contrast enhancement of medical images in general and conjunctival images in particular. Different efforts have been made to this end either by selecting CLAHE parameters that maximize image entropy^16^^,^^17^ or by optimizing them using more elaborate approaches like machine learning^18^^,^^19^ or metaheuristic optimization algorithms.^20^^–^^26^ Nature-inspired optimization algorithms (NIOA) are metaheuristic algorithms derived from natural processes that are used for problem-solving guiding a search process to find near-optimal solutions. Because of their easy implementation, flexibility, and low computational cost, they have been increasingly used to solve a broad range of problems.^27^ Among the large availability of NIOA, the cuckoo search (CS) algorithm^28^ stands out because of (1) its simplicity, its straightforward principle and having very few parameters to tune makes it easy to implement, (2) its robustness, it can handle noisy and uncertain data, and (3) its balance between exploration and exploitation, the combination of Lévy flight with biased random walk guides the search to find the global optima in an efficient way. In fact, it has shown a better performance than other popular NIOA.^29^^,^^30^ Kuran and Kuran^31^ first proposed the use of a multi-objective version of CS to optimize the parameter selection for CLAHE and later showed the added value of this algorithm for disease classification with deep learning.^25^

Despite the growing interest in the assessment of conjunctival vasculature for diabetes and diabetic retinopathy diagnose,^1^ dedicated literature on image processing frameworks for medical purposes is still scarce, especially when compared to the extensive literature available focused on retinal vasculature images preprocessing.^17^^,^^21^^,^^32^^–^^35^ Only a few studies have focused on preprocessing techniques for conjunctival images^36^^,^^37^ and the use of an optimization algorithm for CLAHE implementation in images of the conjunctival vasculature has not been assessed yet. Considering the value of conjunctival vasculature assessment and its prospects for disease detection, it is justified to study dedicated image enhancement techniques for conjunctival images that could further translate into better segmentation and improved disease classification scheme.^21^

The aim of this study is to implement the multi-objective cuckoo search (MOCS) algorithm to tune the parameters of CLAHE for the enhancement of the conjunctival vascular network. Given the poor exposure frequently found in conjunctival images and to ascertain the extension to which this might affect CLAHE performance, the enhancement outcomes are compared for three groups of images with different exposure. The performance of CLAHE with optimized parameter selection is compared to that with fixed parameters to determine if there is an actual improvement.

## Methods

### Image Acquisition

Short video sequences (between one to two seconds) of the bulbar conjunctiva were acquired with a Functional Slit Lamp Biomicroscope (FSLB) consisting of a Huvitz 7000 slit lamp (Huvitz Corp., Anyang-si, Republic of Korea) coupled with a digital camera Canon EOS 60D (Canon Inc., Tokyo, Japan). The angle between the observation system and the illumination system of the slit lamp was set to 45°, and the red-free built-in filter of the slit lamp was used to enhance the structure of the vessels. This digital camera has a CMOS sensor with a maximum resolution of 5.184 × 3.456 pixels (∼17.9 megapixels, pixel size 4.3 × 4.3 µm). Videos were taken using the Movie Crop function available in Canon EOS 60D at a frame rate of 50 fps with an ISO sensitivity of 640. When the Movie Crop function is selected, the operational sensor of the camera is cropped from 5.184 × 3.456 pixels to 640 × 480 pixels resulting in an equivalent of 7.5×. Unlike digital zoom, this magnification maintains the original pixel interval, preserving the original image quality.^38^ Additionally, the slit lamp has five magnification settings, from 6× to 40×, which combined with the magnification of the Movie Crop function results in a range of magnifications from 45× to 300×, allowing us to image the microvessels of the conjunctiva at high resolution and magnification.

The luminosity information of the frames was isolated from chromatic information by converting the recorded sequences from RGB to HSV color space and keeping the value channel (V). Images of the conjunctival vasculature acquired using large magnifications suffer from blinks and eye motion artifacts resulting in defocused frames. To remove those, the sharpness of each frame was estimated by filtering the frame with a Laplacian of Gaussian operator and calculating the variance of the response, where higher variance means more sharpness and better focus.^39^ Then only the most focused frame was chosen for enhancement analysis. All the images were enhanced using CLAHE method with fixed predetermined parameters and CLAHE method with optimized selection of parameters using MOCS algorithm (from now CLAHE and MOCS-CLAHE, respectively).

### Image Exposure Classification

The selection of CLAHE parameters is significantly influenced by the brightness of the input image, because different exposure levels require adjustments of key parameters such as the clip limit and tile size to balance contrast enhancement and noise suppression. Inadequate image exposure, a common issue in clinical practice, can impair the visibility of relevant structures. To address this challenge and evaluate the efficacy of an automated algorithm for optimizing CLAHE parameters, the performance of both CLAHE and MOCS-CLAHE was assessed under varying exposure conditions. After acquisition, images were categorized into three exposure groups: underexposed, correct-exposed, and overexposed. This classification was performed by calculating the average image luminosity (*V_avg_*), defined as the mean intensity in the HSV color space. Images with *V_avg_* ≤ 0.6 were classified as underexposed, those with 0.6 < *V_avg_* ≤ 0.8 as correct-exposed, and those with *V_avg_* >  0.8 as overexposed. These thresholds were selected empirically, considering the specific histogram characteristics of conjunctival images. These images typically exhibit a negatively skewed intensity distribution caused by the dominance of a bright white background, resulting in properly exposed images having an average luminosity above 0.5. The skewed distribution presents unique challenges for contrast enhancement, because underexposed images may obscure critical vascular structures, whereas overexposed images can lead to saturation and loss of detail, highlighting the importance of automating parameter selection for image enhancement. Automated algorithms offer greater consistency and robustness across a wide range of exposure conditions, reducing the variability introduced by manual adjustments.

### Clip Limited Histogram Equalization (CLAHE)

The CLAHE algorithm divides the input image into smaller, non-overlapping contextual regions (i.e., tiles) in which local histogram equalizations are independently performed.^40^ To prevent overamplification, especially in the most homogeneous regions, the original histogram of each tile is clipped to a predefined limit (i.e., clip limit), and the values exceeding this limit are equally redistributed across all the gray level values before the transformation function is applied.^41^ This step restricts contrast enhancement and decreases noise amplification, with a smaller clip limit yielding lower contrast enhancement and reduced noise amplification in the enhanced image. Using the clipped and redistributed histogram, the cumulative distribution function is calculated and used to obtain a transformation function, which defines how original input pixels values are remapped to new output values in each tile. After applying the transformation function to all the tiles, they are rejoined using a bilinear interpolation which helps to minimize artifacts, such as blocky transitions or edge discontinuities at the boundaries between tiles.^42^

The contrast transfer function used in CLAHE can be computed with different probability density functions, which define the shape of the output histogram. In this work, CLAHE was performed using a Rayleigh distribution due to its ability to model smooth brightness transitions, making it particularly suitable for medical images.^43^ The shape of the Rayleigh distribution is governed by the parameter α, which directly influences the brightness and performance of the enhanced image.

Altogether, CLAHE requires the optimization of three key parameters for optimal performance: the clip limit, which controls how much contrast is enhanced; the number of tiles, which affects the granularity of local equalization; and the α parameter of the Rayleigh distribution, which adjusts the image brightness. The choice of these parameters is influenced by image characteristics, such as brightness distribution, texture, and noise level. For example, a higher clip limit may over enhance noisy regions, while too many small tiles can cause excessive local variation in contrast. Similarly, adjusting the α parameter alters the balance between contrast and brightness. Selecting optimal parameters is crucial, as improper values can either amplify noise or fail to enhance image contrast sufficiently, impairing the visibility of important features. These challenges are particularly relevant for medical images, where accurate visualization of structures is essential for diagnosis.

### Multi-Objective Cuckoo Search Algorithm (MOCS)

The cuckoo search algorithm is a meta-heuristic algorithm used to solve optimization problems that finds adequate solutions in a fast and efficient way.^28^ This algorithm is inspired by the nature of the parasitic behavior of some cuckoo birds. These brood parasite birds don’t build their own nest; instead they lay their eggs in a host bird nest to increase their survival rates and population. Because only the best cuckoo eggs will survive, the cuckoo must mimic the pattern and colors of the host eggs to reduce the likelihood of the cuckoo egg being discovered. The host bird can discover the cuckoo egg with a probability $P_{a}\in \left[0,\;1\right]$. If this happens, the host bird will throw the intruder away or abandon the nest to build a new one.

Translated to the numerical optimization problem, each nest is a population, each egg of a nest represents a solution, and the cuckoo egg is a new solution. During an iterative process, one egg of each nest is replaced with a new solution. The new solution will be kept for the next iteration only if it is better than the existing ones and if the random probability of discovery <*P_a_*. For the original cuckoo search algorithm implementation, there are three rules that have to be met: (1) each cuckoo bird only lays one egg per iteration in a randomly chosen nest, (2) only the best quality eggs pass to the next iteration, and (3) the number of available nests is fixed, and the cuckoo egg is discovered with a probability $P_{a}\in \left[0,\;1\right]$. The cuckoo search algorithm finds the optimal solutions by balancing exploitation (local search) via Lévy flight random walk and exploration (broad search) by biased/selective random walk. While the objective of the Lévy flight random walk is to generate solutions similar to the best solutions, the biased/selective random walk generates solutions far from the best solution, preventing the algorithm from being stuck in a local optimum and increasing the diversity of the solutions.

Typically, the quality of the solutions is assessed using an objective metric as fitness function. However, the use of a single metric (single objective) is not always adequate, because in many optimization problems, the desired solution needs to satisfy multiple criteria (multi-objective) that are in conflict. For example, for contrast enhancement we want to maximize contrast but also limit noise amplification. Conversely to a single objective solution, in a multi-objective problem there is not a single optimal solution that maximizes one single objective but a set of optimal solutions that represent a compromise between multiple conflicting objectives. This set of solutions is known as the Pareto front and it is sorted using a nondominated approach and crowding distances, meaning that one fitness function is not more important than the other, instead a balanced solution is desirable. From the Pareto front, the final solution is chosen as the middle one from the front because it represents a balanced tradeoff between two or more conflicting objectives (in this case, they would be contrast and noise).^44^ A variant of the cuckoo search algorithm to solve multi-objective problems using Pareto front is proposed by Yang and Deb.^45^

For the CLAHE problem the variables that are meant to be optimized are the number of tiles, the clip limit, and the α of the Rayleigh distribution. To evaluate the quality of the solutions, CLAHE is performed with each set of solutions and the resulting image assessed with two fitness functions. These two objectives have been chosen according to the premise of enhancing image contrast while preventing noise amplification. The first function is the contrast obtained from the gray level co-occurrence matrix (GLCM), which is meant to be maximized. The GLCM represents the probability of finding two gray levels at a specific distance and direction in a gray-scale image.^46^ From this co-occurrence matrix various statistical descriptors can be calculated. Particularly, the GLCM contrast measures the local intensity variations in the image, and it is defined as:

(1) $$\begin{array}{c} \mathrm{Contrast}=\sum_{i,j}{\left|i-j\right|}^{2}p\left(i,j\right)\; \end{array}$$

where *p*_*i*,*j*_ is the (*i*, *j*)^th^ entry of the normalized GLCM of the image.

The second fitness function is meant to be minimized as it estimates noise amplification with the fast noise variance estimation (FNVE) method.^47^ In FNVE method a noise estimation operator (*N*) is calculated as the difference between two 3 × 3 Laplacian operator approximations and convolved with image *I (x, y)*. The variance of the output is calculated at each pixel, and it gives an estimate of $36\sigma_{n}^{2}$ that is averaged over *I* to obtain an estimate of the noise variance σ_*n*_. The variance estimate is computed as

(2) $$\begin{array}{c} \sigma_{n}=\sqrt{\frac{\pi }{2}}\frac{1}{6\left(W-2\right)\left(H-2\right)}\sum_{I}\left|I\left(x,y\right)*N\right|\; \end{array}$$

where *W* and *H* are the width and height of the image, respectively, and *N* is the noise estimator operator.

**Figure 1.**
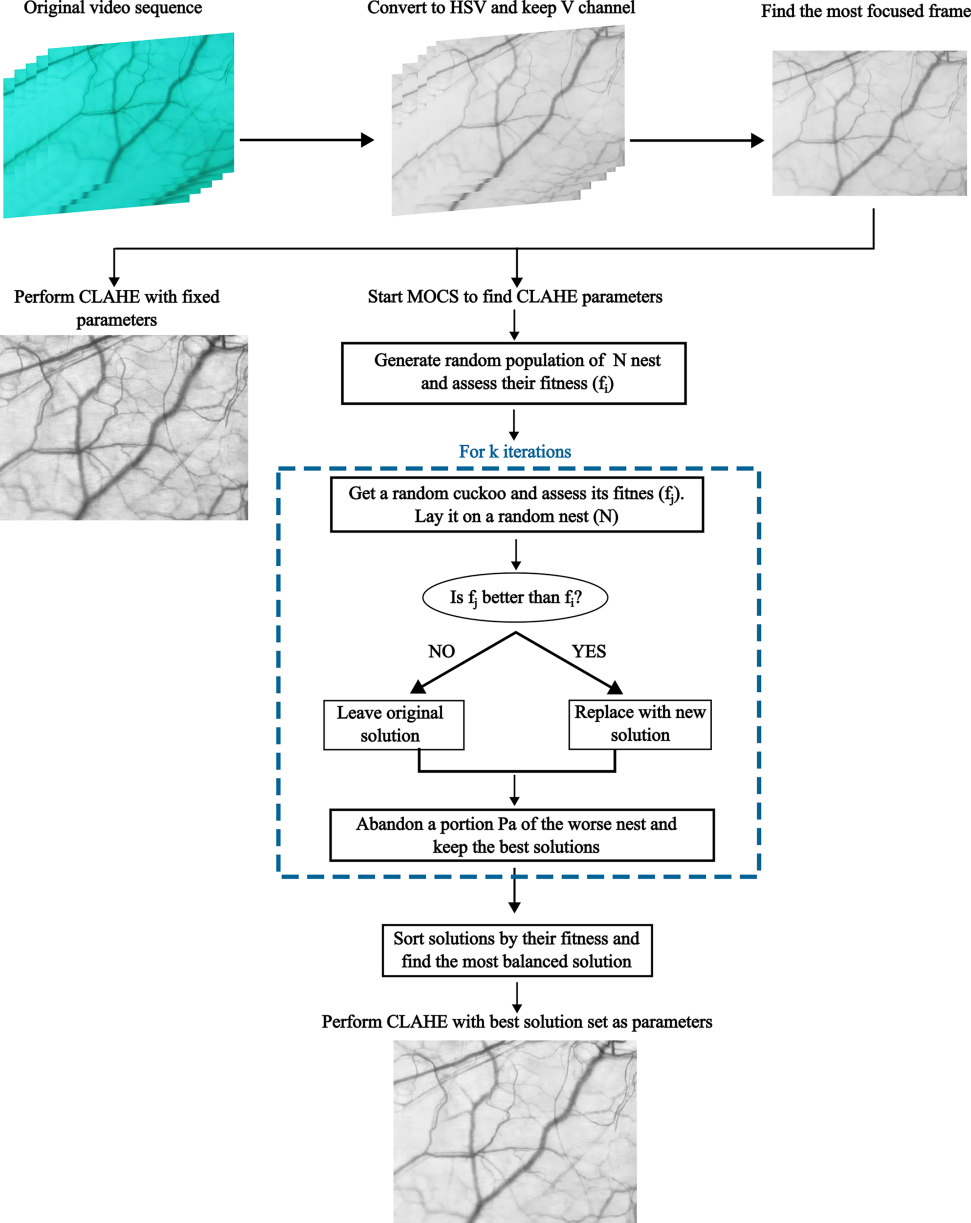
Flowchart showing the image processing pipeline and the basic steps of MOCS algorithm.

Figure 1 depicts the entire pipeline of the different image processing steps, from image conversion and frame selection to the basic steps of MOCS algorithm. The code for MOCS algorithm can be found at github.com/ClaraLlorens/MOCS-CLAHE.

### Quantitative Performance Assessment

To quantitatively and objectively evaluate the performance of CLAHE and MOCS-CLAHE peak signal-to-noise ratio (PSNR), structural similarity index measure (SSIM), and natural image quality evaluator (NIQE) were used as evaluation metrics.

The PSNR quantifies the corrupting noise of the enhanced image with respect to the original image, where higher PSNR corresponds to better quality. It calculates the ratio between the maximum value of a signal and the value of the noise of the enhanced image by the mean square error (MSE):

(3) $$\begin{array}{ccc} & & \mathrm{PSNR}=10\cdot log_{10}\left(\frac{{\left(L-1\right)}^{2}}{MSE}\right),\\ & & \mathrm{MSE}=\frac{1}{M\times N}\sum_{i=1}^{M}\sum_{j=1}^{N}\lceil I\left(i,\;j\right)-I_{e}\left(i,\;j\right)\rceil^{2}\; \end{array}$$

where *L* is the number of gray levels, *M × N* the number of pixels, *I*(*i*,  *j*) the original image, and *I_e_*(*i*, *j*) the enhanced image.

The SSIM quantifies the perceived structural degradation of the enhanced image with respect to the original image. The higher the SSIM is the better the enhanced image preserves the geometric features. Its calculation is given by

(4) $$\begin{array}{ccc} \mathrm{SSIM} & = & \frac{\left(2\mu_{I}\mu_{Ie}+C_{1}\right)\left(2\sigma_{I\;Ie}+C_{2}\right)}{\left(\mu_{I}^{2}+\mu_{Ie}^{2}+C1\right)\left(\sigma_{I}^{2}+\sigma_{Ie}^{2}+C_{2}\right)},\\ C_{1} & = & {\left(K_{1}L\right)}^{2},\;C_{2}={\left(K_{2}L\right)}^{2}\; \end{array}$$

where μ_*I*_ and μ_*Ie*_ are, respectively, the mean of the original and enhanced image, σ_*I*_ and σ_*Ie*_, respectively, the variance of the original and enhanced image and σ_*I* *Ie*_ the co variance between the original and the enhanced image. *K*_1_ and *K*_2_ are constant with values 0.01 and 0.03, respectively.

The NIQE is a nonreference image quality metric that uses natural scene statistics model features to calculate the distance to the features from the problem image and give a score quality.^48^ The smaller the NIQE the better the perceptual quality of the image.

### Statistical Analysis

Descriptive statistics are expressed as mean ± standard deviation (SD). Because the data did not follow a normal distribution, as assessed by Shapiro-Wilk test, nonparametric inference tests were used for statistical comparisons. The Friedman test, a nonparametric alternative to repeated measures analysis of variance, was used to compare contrast and noise across the original, CLAHE-enhanced, and MOCS-CLAHE-enhanced images. The Wilcoxon signed-rank test was used to compare paired performance metrics between CLAHE and MOCS-CLAHE. The significance threshold was set at *P* < 0.05 and adjusted using Bonferroni correction to account for multiple comparisons and control type I error.

## Results

**Figure 2.**
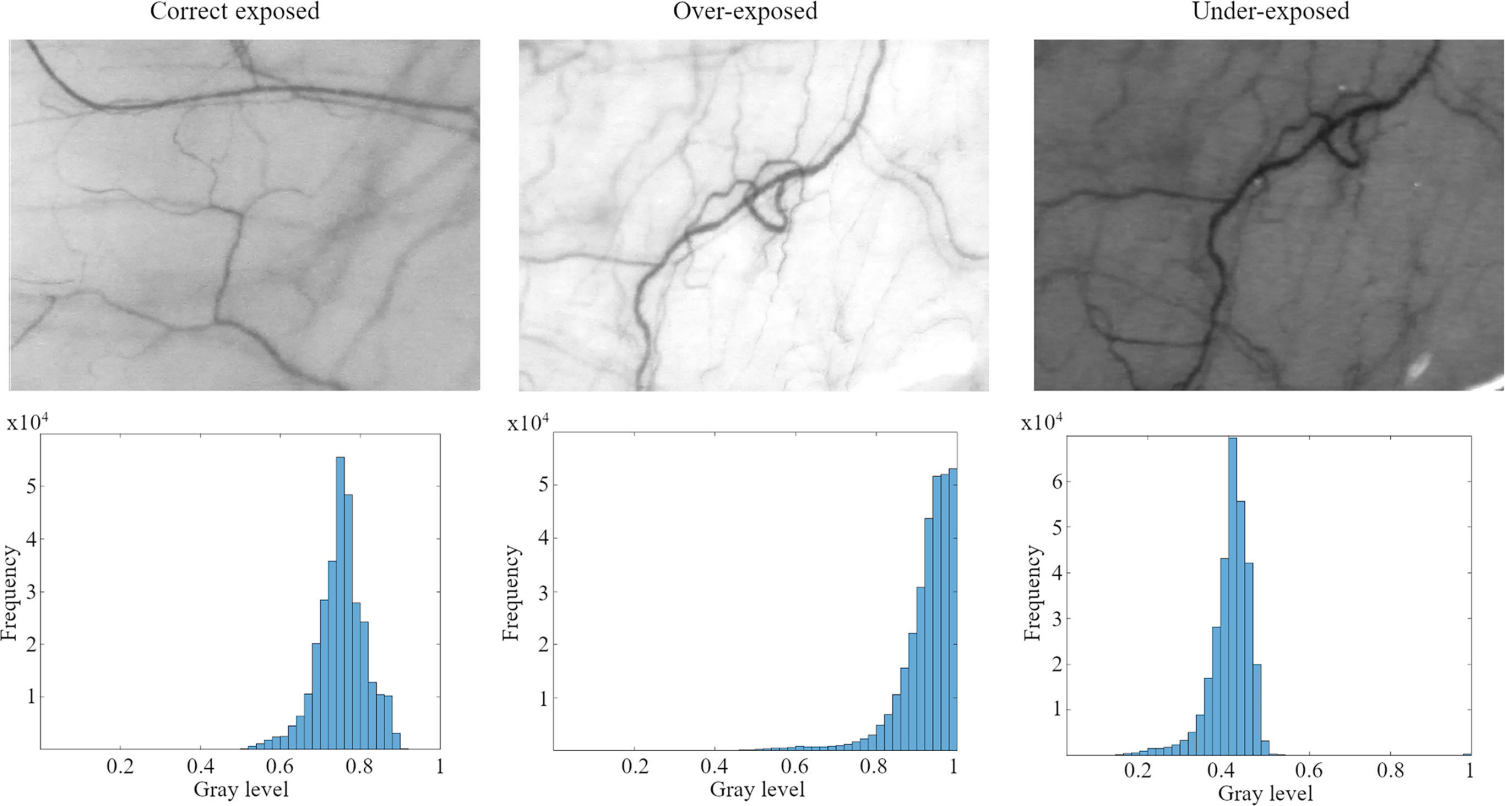
Examples of three conjunctival images (*upper row*) with their corresponding histogram counts (*lower row*) with different exposure.

A total of 166 images of the temporal bulbar conjunctiva were acquired using different magnification settings. Eleven of those images were discarded because of bad focus, high movement, or lack of vessels in the imaged area. Forty-three images were allocated to the correct exposure group, 60 to the overexposed group and 52 to the underexposed group. Figure 2 shows an example of an image of each exposure group with their corresponding histograms.

**Figure 3.**
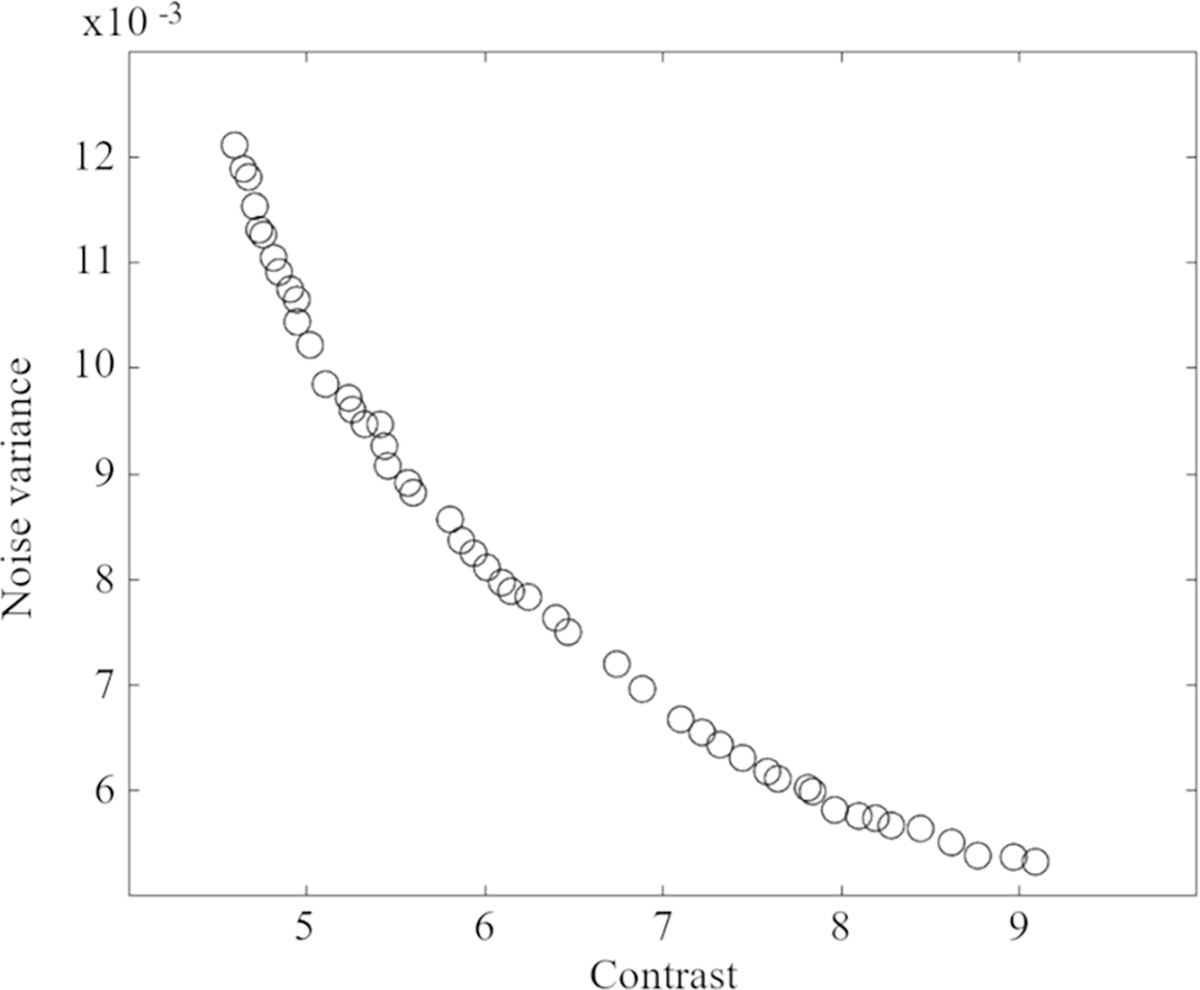
Example of the fitness of the Pareto front set of solutions that represents a trade-off between contrast enhancement and noise amplification. Noise variance is calculated using the fast noise variance estimation method (see Equation 2) and the contrast is calculated from the gray-level co-occurrence matrix (see Equation 1).

All images were processed using both enhancement approaches (i.e., CLAHE and MOCS-CLAHE). For CLAHE the number of tiles was set to 8 × 8, the clip limit to 0.01 and the α parameter of the Rayleigh distribution to 0.6. These parameters were empirically chosen to maximize the image entropy of the enhanced images. The parameters used for MOCS-CLAHE are shown in Table 1. The upper and lower bounds were chosen empirically by testing first in a subset of images and making sure that the range was wide enough not to miss optimal solutions while keeping a restricted space of search. Figure 3 shows an example of the fitness for the different solutions in the Pareto front that represents the tradeoff being optimized.

**Figure 4.**
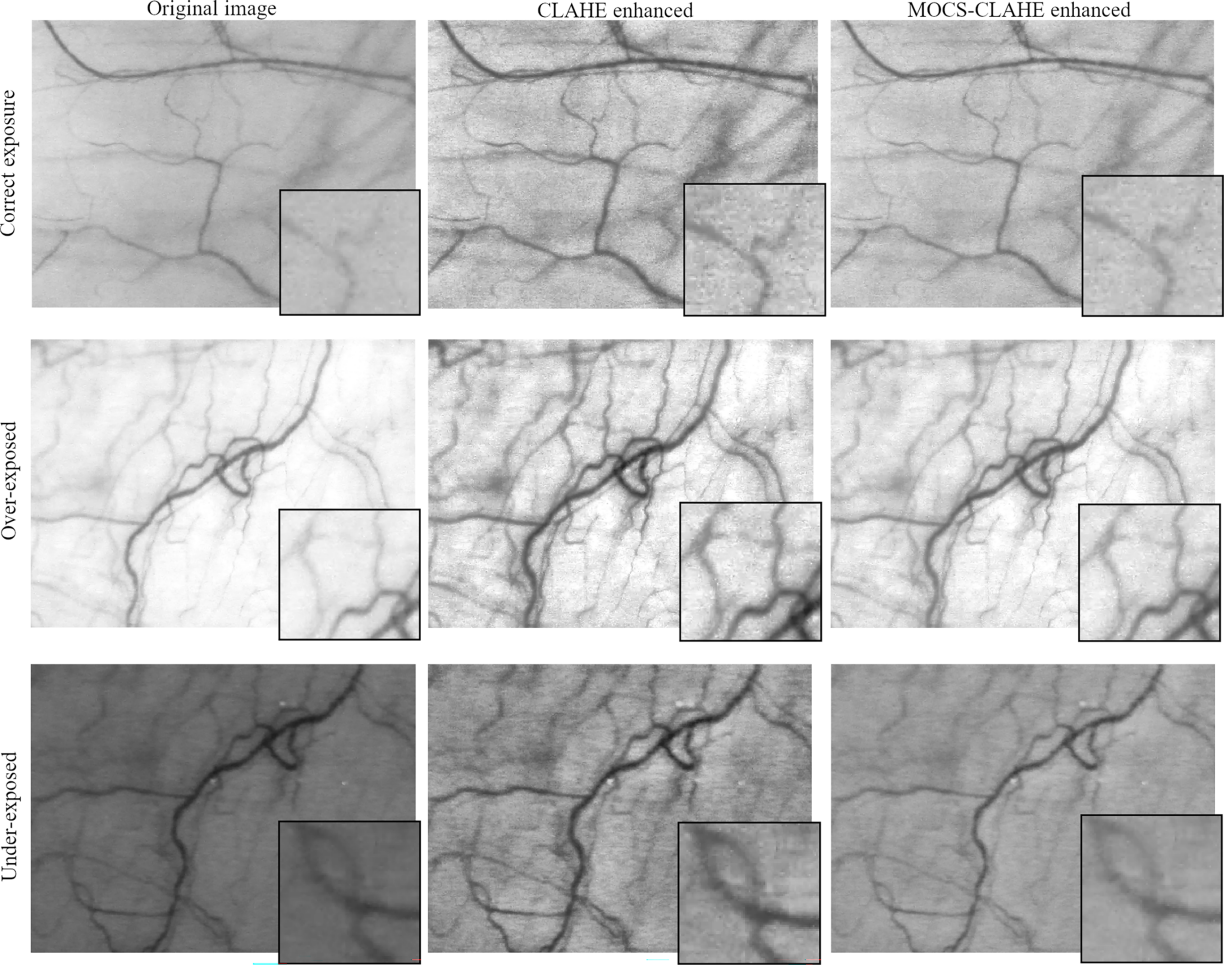
Visual comparison with zoomed regions of the performance of CLAHE and MOCS-CLAHE in a correct-exposed image (*upper row*), overexposed image (*middle row*), and underexposed image (*bottom row*).

A visual comparison between CLAHE and MOCS-CLAHE enhancements is shown in Figure 4 where three random examples of each of the exposure groups have been chosen to illustrate the qualitative differences between both methods of enhancement.

**Table 1.** Parameters for the Multi-Objective Cuckoo Search Algorithm

Parameter	Value
Population size	50
Number of iterations	20
Probability of survival (100% − P_a_)	25%
Number of dimensions	4^*^
Upper bounds	
Number of horizontal tiles	12
Number of vertical tiles	12
Clip limit	0.01
α	0.9
Lower bounds	
Number of horizontal tiles	4
Number of vertical tiles	4
Clip limit	0.001
α	0.5

* Number of horizontal and vertical tiles, clip limit and α of Rayleigh distribution.

Table 2 shows the contrast and the FNVE, respectively, for each group of exposure. The contrast of the original images was 0.086 ± 0.018, 0.043 ± 0.033, and 0.070 ± 0.020 for correct-, over- and underexposed images, respectively. Each enhancement approach significantly increased the contrast of the original images (*P* < 0.001, Wilcoxon Rank test). Although contrast enhancement was higher with CLAHE approach for the three groups (Table 2, *P* < 0.001, Wilcoxon Rank test), MOCS-CLAHE significantly reduced noise amplification compared to CLAHE (Table 2, *P* < 0.001, Wilcoxon Rank test).

**Table 2.** Image Contrast and Noise (FNVE) for the Original Image and the Enhanced Image With CLAHE and MOCS-CLAHE

	Image Contrast			FNVE		
Mean ± SD	Original Image	CLAHE	MOCS-CLAHE	Original Image	CLAHE	MOCS-CLAHE
Correct exposure	0.086 ± 0.018	0.21 ± 0.030	0.15 ± 0.060	0.0040 ± 0.00066	0.010 ± 0.0015	0.0065 ± 0.0029
Over-exposed	0.043 ± 0.033	0.14 ± 0.066	0.077 ± 0.058	0.0031 ± 0.0015	0.0083 ± 0.0028	0.0048 ± 0.0023
Under-exposed	0.070 ± 0.020	0.18 ± 0.02	0.13 ± 0.040	0.0029 ± 0.00035	0.0073 ± 0.00078	0.0043 ± 0.0016

**Figure 5.**
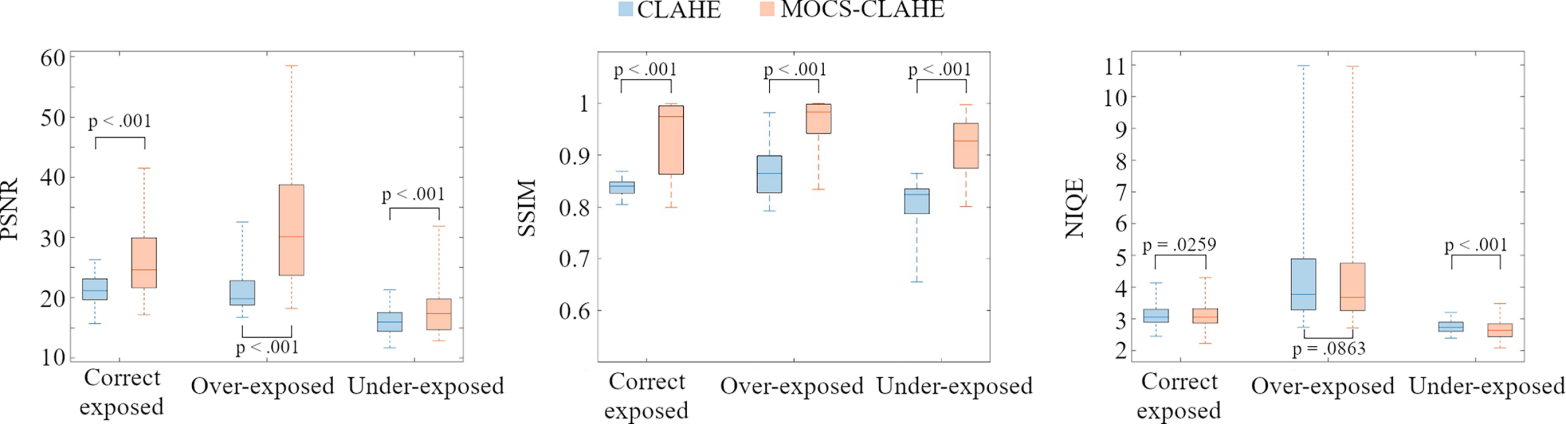
Box-plots showing the quantitative image quality performance of CLAHE and MOCS-CLAHE for correct-exposed, overexposed, and underexposed images. The *P* values between CLAHE and MOCS-CLAHE are obtained from the matched Wilcoxon-signed rank test.

Image quality, assessed with quantitative metrics (i.e., SSIM, PSNR and NIQE), of the enhanced images with both approaches is shown in the box plots of Figure 5 and Table 3. For all cases, MOCS-CLAHE showed significantly better performance, with higher PSNR and SSIM values and lower NIQE outcomes.

**Table 3.** Quantitative Image Quality Metrics for Enhanced Images

	PSNR			SSIM			NIQE		
Mean ± SD	CLAHE	MOCS-CLAHE	*P* ^*^	CLAHE	MOCS-CLAHE	*P* ^*^	CLAHE	MOCS-CLAHE	*P* ^*^
Correct-exposure	21.30 ± 2.38	26.06 ± 5.44	<0.001	0.84 ± 0.016	0.94 ± 0.069	<0.001	3.10 ± 0.40	3.07 ± 0.45	0.0259
Overexposed	20.93 ± 3.19	32.25 ± 10.11	<0.001	0.87 ± 0.044	0.97 ± 0.040	<0.001	4.45 ± 1.86	4.41 ± 1.90	0.0863
Underexposed	16.20 ± 2.32	18.10 ± 4.00	<0.001	0.81 ± 0.044	0.92 ± 0.055	<0.001	2.76 ± 0.21	2.65 ± 0.29	<0.001

* Matched Wilcoxon-signed rank test.

## Discussion

In this work we have implemented MOCS algorithm to optimize the parameter selection of CLAHE and enhance the contrast and visibility of the conjunctival vascular network. The performance of CLAHE using optimized parameter selection (i.e., MOCS-CLAHE) was compared against its performance with fixed parameters (i.e., CLAHE) in three groups of images with different image exposure (i.e., underexposed, correct-exposed and overexposed images). Besides a qualitative visual comparison, we have performed a quantitative comparison using SSIM, PSNR and NIQE evaluation metrics. These results show that MOCS-CLAHE enhances the contrast and visibility of the conjunctival vessels while keeping noise amplification to a minimum and resulting in an overall higher quality image than when enhanced with CLAHE (Fig. 5; Table 3).

The optimal CLAHE parameters can vary significantly from one type of image to another, for example the parameters that better enhance an MR image might not be suitable for an ultrasound image because their frequency, gray-level distribution, or speckle content is very different. When imaging the vascular network of the conjunctiva, it is common to acquire images at different magnifications or with different instruments and thus the information content in each image varies considerably. Additionally, given the high reflective nature of the conjunctiva and the use of high magnifications, the amount of light at the image plane (camera sensor) is very sensitive to small movements that happen during the acquisition. This translates into images either over- or underexposed, which accentuates the need of optimizing CLAHE parameters for each individual image because they not only depend on the type of object being imaged but also on the illumination. In this work we have used Rayleigh distribution to design the transfer function of CLAHE. The α parameter of the Rayleigh distribution controls its shape and will dictate the luminosity of the enhanced image, so its optimal value will also depend on the original exposure of the image. In the study by Jintasuttisak and Intajag,^35^ the need for adapting CLAHE parameters to image exposure is shown. To highlight the advantage of using an optimization algorithm for parameter selection, we have assessed the performance of CLAHE and MOCS-CLAHE in images with poor exposure, and the results show that MOCS-CLAHE still works better under images with poor illumination.

The need of proper selection of CLAHE parameters^15^ and the advantages of metaheuristic algorithms for solving optimization problems has fostered many works on CLAHE parameter optimization using NIOA for medical image preprocessing.^20^^–^^25^ Among all the available NIOA, we chose to use the cuckoo search algorithm because of its easy implementation; it only has one parameter to be tuned, which is the probability of discovery; it can be used to solve a wide range of different problems; and it has a good balance between global and local search. Moreover, its efficiency in CLAHE parameters optimization was previously demonstrated,^31^ and its performance has shown to be superior compared to other image-based contrast enhancement algorithms.^29^ Another approach would be to use machine learning-based image enhancement algorithms,^18^^,^^19^ but these algorithms need large data sets to be trained for a particular task, and they are computationally expensive and thus not suitable for real-time applications. The proposed algorithm can be used to enhance conjunctival images regardless of the instrumentation used to acquire the images and without the need of additional adjustments for each data set.

The MOCS algorithm uses more than one metric to assess the fitness of the solutions.^45^ For conjunctival vasculature images, the aim is to enhance the visibility and contrast of the vascular structures. However, increasing the contrast of the images comes with the cost of adding noise that, if not considered, will undermine the quality of the enhanced image, as can be seen in the CLAHE results of Figure 4. Then, the goal is to increase contrast and limit noise amplification. Because these two premises are in conflict, it is not possible to maximize one and the other; instead we need to find a compromise solution consisting of enough contrast enhancement without too much noise. Thus it becomes clear that to achieve the best quality enhancement, two (or more) objective metrics need to be used in the optimization process. MOCS algorithms have shown promising results in solving complex optimization problems. These approaches extend the single-objective cuckoo search to handle multiple objectives simultaneously.^45^ MOCS algorithms often incorporate non-dominated sorting and archiving techniques to efficiently identify Pareto-optimal solutions.^49^ For this work, image contrast and noise were chosen as the objective metrics to select MOCS-CLAHE parameters, intending to maximize the first and minimize the second. On the other hand, fixed CLAHE parameters were chosen by assessing the clip-limit and block size versus the image entropy in a subset of images with the goal of maximizing the contrast, similarly as proposed by Min et al.^50^ For CLAHE, those same parameters were used for all the images. The advantage of using a multi-objective solution is seen in Table 2 and Figure 4, whereas the CLAHE method increases the contrast even more than MOCS-CLAHE, it generates significantly more noise resulting in an image with overall worse quality as assessed by the significantly lower SSIM and PSNR and higher NIQE. MOCS-CLAHE keeps better structural similarity with the original image compared to CLAHE and this is of upmost importance for the assessment of the conjunctival vasculature and further image segmentation.

In this work CLAHE parameters optimization is computed in a single frame rather than in all the frames of the sequence, assuming minimal variations in image content and illumination given the short duration of the video sequences (i.e., one to two seconds). In further steps, the obtained parameters for the most focused frame are used to enhance the remaining frames of the sequence that are further registered to obtain a higher quality image. It is important to perform image enhancement before image registration because that will enable to find more reliable landmarks for a robust registration. Despite the recognized value of CLAHE enhancement for retinal image classification and retinal vessels segmentation,^17^^,^^34^ only a few studies have focused on CLAHE preprocessing techniques for conjunctival images^36^^,^^37^ and the use of an optimization algorithm for CLAHE implementation in images of the conjunctival vasculature has not been assessed yet.

This study has a few limitations that should be acknowledged. First, the enhancement process was applied only to grayscale images, limiting the approach's applicability to color images, where preserving chromatic information is essential to prevent color distortion. To study the structure of the vessels color information is not needed, but to generalize this approach for different types of analyses where color can add valuable information an approach to isolate and enhance the luminosity of the image and then recombine it with its chromatic information to avoid color alterations should be applied. Additionally, despite metaheuristic algorithms having faster execution times than exact algorithms, they were not initially designed for real-time applications and their convergence speed when dealing with large problems can be slow, reducing the algorithm feasibility in real-time applications. In this work the computational time has not been optimized, and the time required for the optimization is still relatively high, posing challenges for real-time applications. Currently, the approximate processing time is about 50 seconds per image using an Intel Core i7 12700H processor with 32 GB of RAM, and no GPU acceleration. The computational time could be reduced using strategies like parameter control, hybrid approaches or parallel processing, broadening cuckoo-search algorithm to real-time applications. This could be potentially applicable to the assessment of the hemodynamic properties of the conjunctiva with real-time imaging systems. Last, the empirical thresholds for exposure classification may require further validation across different datasets and imaging conditions to ensure broader applicability. Future research should focus on addressing the limitations identified in this study.

In conclusion, this work shows the advantages of using the MOCS metaheuristic algorithm for the enhancement of the conjunctival vascular network. It proved to be an efficient method to estimate CLAHE parameters and provide high quality images with increased contrast and low noise content. To the best of our knowledge, this is the first study to apply a metaheuristic algorithm to enhance the vascular structure of the ocular conjunctiva. This automation has the potential to improve the reliability of conjunctival FSLB imaging in the assessment of morphological and structural changes of conjunctival vessels in diabetes and to advance in the understanding of diabetic retinopathy physiopathology and how it relates to the vascular alterations in the conjunctiva. However, it is expected to be useful to study the conjunctival vascular network not only in diabetes but also in other systemic diseases with an associated vasculophathy. Despite this algorithm has been developed and implemented with FSLB images, given the flexibility and adaptability of MOCS algorithm it is anticipated that other conjunctival imaging technologies could also benefit from it by adjusting MOCS parameters. Extending the application of the algorithm to other types of imaging techniques could provide insights into its generalizability. The use of optimization algorithms for image enhancement has proven to be valuable for further image processing steps such as segmentation and image classification.^25^^,^^34^^,^^51^ Thus it is expected that the MOCS-CLAHE enhanced images will allow better image segmentation with the consequent improvement in the assessment of vascular parameters and image classification for diabetes and diabetic retinopathy. Investigating the algorithm's impact on key clinical tasks, such as vessel segmentation accuracy and disease classification, is also crucial to fully assessing its utility. Furthermore, future studies should explore integrating the algorithm into real-time imaging systems to enhance diagnostic workflows in clinical settings.
